# Cut-Off Value of Medial Meniscal Extrusion for Knee Pain

**DOI:** 10.1155/2017/6793026

**Published:** 2017-04-16

**Authors:** Hiroaki Kijima, Naohisa Miyakoshi, Yuji Kasukawa, Yoshinori Ishikawa, Hayato Kinoshita, Kentaro Ohuchi, Masazumi Suzuki, Nozomi Kaga, Chie Sato, Shuichi Chida, Yoichi Shimada

**Affiliations:** ^1^Department of Orthopedic Surgery, Akita University Graduate School of Medicine, 1-1-1 Hondo, Akita 010-8543, Japan; ^2^Akita Sports, Arthroscopy, and Knee Group (ASAKG), 1-1-1 Hondo, Akita 010-8543, Japan

## Abstract

*Purpose*. Medial meniscal extrusion (MME) has attracted attention as an index of knee pain in conjunction with clinical symptoms that could be more useful than the diagnosis of knee osteoarthritis on X-ray. However, the size of MME that would cause knee pain has not been clarified. The aim of the present study was to investigate the cut-off value of MME for knee pain.* Methods*. A total of 318 knees were evaluated. The presence of current or past knee pain was confirmed by interview. Next, MME was measured using vertical sonographic images of the medial joint spaces during weightbearing.* Results*. Overall, 71 knees were painful (P-group), and 247 knees were not (N-group). MME was 5.9 ± 1.8 mm in the P-group and 2.9 ± 1.5 mm in the N-group (*P* < 0.0001). Analysis of the receiver operating characteristic curve showed that the cut-off value of MME for knee pain was 4.3 mm, with sensitivity of 0.8451 and specificity of 0.8502. In addition, 64% of knees without pain cases at the time of examination whose MME exceeded this cut-off value had past knee pain.* Conclusions*. The sensitivity and specificity of MME for knee pain were very high with a cut-off value of 4.3 mm.

## 1. Introduction

Knees diagnosed as having osteoarthritis on X-ray or magnetic resonance imaging (MRI) may not have pain. Thus, there is asymptomatic knee osteoarthritis. In addition, there are knees with severe pain and knees with only slight pain, even though they have similar imaging findings. Furthermore, even if imaging indicates knee osteoarthritis, knees without pain are rarely treated.

In recent years, many large-scale epidemiological investigations of knee osteoarthritis have been performed to develop guidelines for the diagnosis and treatment of knee osteoarthritis in order to prevent locomotive syndrome and extend healthy life expectancy, as well as standardize treatment policy [1–4]. Most diagnoses are made based on joint space narrowing or osteophyte formation on X-ray or cartilage evaluation on MRI [5], but, as noted above, these findings on X-ray images or MRI do not correlate with the degree of pain, which is the target of the treatment. Therefore, the results provided by this approach differ from epidemiological investigations of symptomatic knee osteoarthritis targeted for treatment, because imaging investigations include a considerable number of asymptomatic knee osteoarthritis cases.

It is necessary to perform epidemiological investigations of symptomatic knee osteoarthritis to standardize the treatment of knee osteoarthritis and to develop a treatment guideline for knee osteoarthritis. To that end, an index related to the degree of pain of knee osteoarthritis is necessary.

In recent years, two papers have reported that medial meniscal extrusion (MME), which can be measured on ultrasonography, is related to the degree of knee pain [6, 7]. Furthermore, MME has attracted attention as an index related to the clinical symptoms of knee osteoarthritis, unlike the X-ray diagnosis of knee osteoarthritis, which does not relate to the degree of knee pain.

MME is defined as deviation of the medial meniscus medially from the joint. Kenny reported for the first time in 1997 that MME represented decreased meniscal function [8]. Many reports demonstrated that MME participated in the progress of knee osteoarthritis, and it was shown that MME reflected cartilage damage more subtly than X-ray findings [9].

On the other hand, pain in the medial joint space during weightbearing is a representative symptom of knee osteoarthritis, but the cause of the pain has not been clarified. It has been confirmed that MME is greater during weightbearing than during nonweightbearing [10]. The soft tissues around the medial joint space may be stretched by this phenomenon, which may cause the pain of knee osteoarthritis through the affected mechanical receptors. However, the amount of displacement needed to cause pain has not been investigated. If this were to be clarified, epidemiological investigations of knee osteoarthritis that could become symptomatic could be easily performed by ultrasonography.

Therefore, the purpose of this study was to determine the cut-off value of MME for knee pain.

## 2. Materials and Methods

The bilateral knees (318 knees) of 159 members of the general population who underwent medical examinations in an area where the birthrate is falling and the population is aging were examined. The subjects' average age was 70 years (30–87 years); there were 154 males and 164 females. The subjects lived in a snowy agricultural area of the northeast district of Japan. In this area, medical examinations of the subjects are performed annually.

First, a questionnaire was used to check whether the participants had knee pain at the time of the examination or in the past. In this study, where the pain was located in the knee was not checked. Then, on ultrasonographic vertical images of the medial joint space in extended knees during weightbearing, the displacement length from the medial tibial condyle to the deep layer of the medial collateral ligament of the medial meniscus was measured by ultrasound examination (ACUSON S1000, Siemens Medical Solutions USA, Inc., Ultrasound Division, Mountain View, CA) to evaluate MME. This method of measurement for MME is the same as that of Kawaguchi et al. [10] and of many reports [6, 7]. Since the intraclass correlation coefficients of this method are 0.990 for intraobserver correlation and 0.989 for interobserver correlation [11], it is not necessary to check the intraclass correlation coefficients for MME evaluation with ultrasonography in our institution.

A receiver operating characteristic (ROC) curve for the relationship between pain and the amount of MME was created, and the cut-off value of MME for knee pain was determined as the point of maximal sensitivity and specificity.

Furthermore, the rate of knees with past knee pain without current pain that had MME beyond the cut-off value was compared with that of knees without current pain that had MME less than the cut-off value. The rates of knees with pain in the past were compared between the 2 groups with and without MME beyond the cut-off value using the chi-squared test. Significance was set at *P* < 0.05.

The approval for this study was given by the institutional review board, and an informed consent was obtained from each participant.

## 3. Results

**Figure 1:**
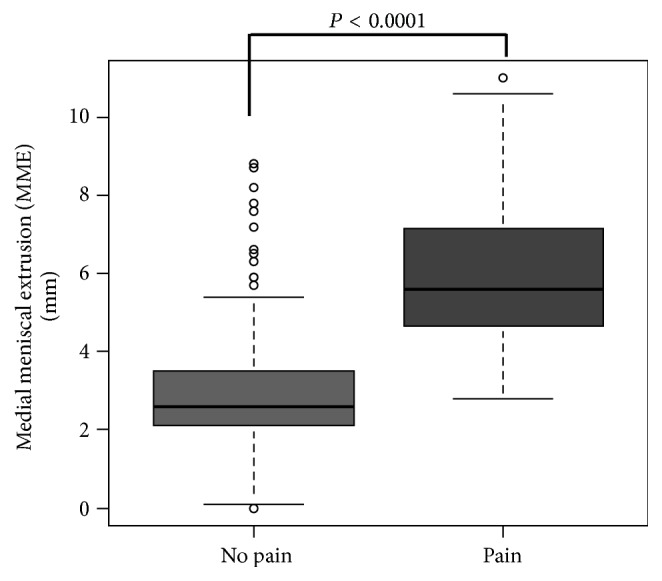
The relationship between medial meniscal extrusion (MME) and knee pain. MME is significantly greater in the P-group (5.9 ± 1.8 mm) than in the N-group (2.9 ± 1.5 mm; Student's* t*-test, *P* < 0.0001).

The P-group had 26 male knees and 45 female knees, and the N-group had 129 male knees and 118 female knees. A total of 71 knees were painful (P-group) and 247 were not (N-group). MME was significantly greater in the P-group (5.9 ± 1.8 mm) than in the N-group (2.9 ± 1.5 mm; Student's* t*-test, *P* < 0.0001) (Figure 1).

**Figure 2:**
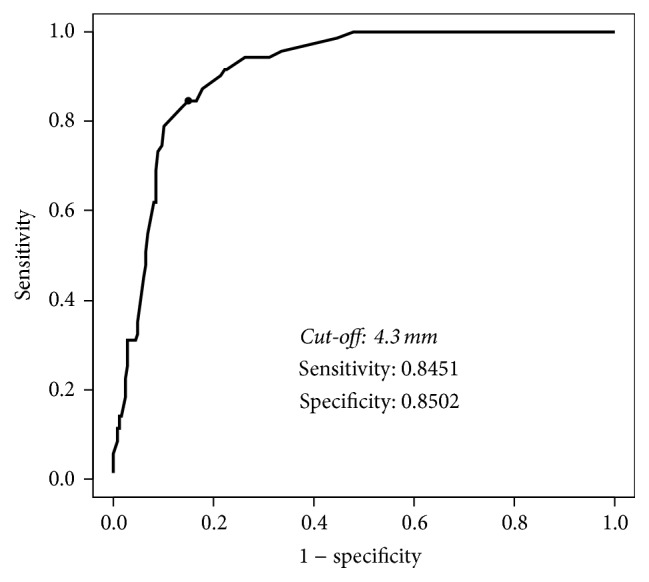
Receiver operating characteristic (ROC) curve of the amount of medial meniscal extrusion (MME) for knee pain. The cut-off value of MME for knee pain is 4.3 mm, with sensitivity of 0.8451 and specificity of 0.8502.

The MME cut-off value for knee pain was 4.3 mm on ROC curve analysis, with sensitivity of 0.8451 and specificity of 0.8502 (Figure 2). Among the knees without current pain but with an MME beyond the cut-off value, 62% had a past history of knee pain.

**Figure 3:**
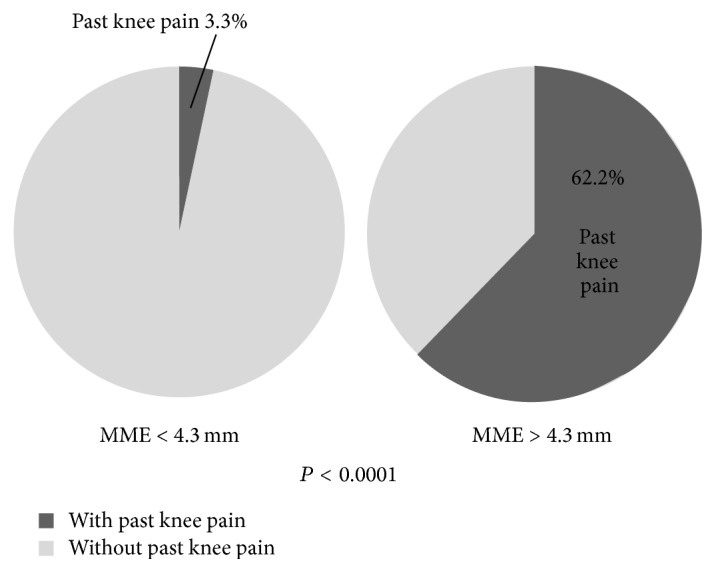
Rate of past knee pain in the N-group. In the N-group, the rate of knees with pain in the past among the knees with MME greater than 4.3 mm is significantly larger than that of knees with MME less than 4.3 mm.

In the N-group, the rate of knees with past knee pain was significantly higher among the knees with MME greater than 4.3 mm than that among the knees with MME less than 4.3 mm (chi-square test, *P* < 0.0001) (Figure 3).

## 4. Discussion

In this study, the presence of knee pain in the general population and the amount of MME were investigated using ultrasonography. When the MME cut-off value for knee pain was 4.3 mm based on the ROC curve analysis, the sensitivity and specificity were very high.

In the current diagnosis of early osteoarthritis of the knee, MME > 3 mm is one of the standard indices [12, 13]. In the present study, the MME cut-off value for pain was 4.3 mm, which is not very different from the 3.0 mm used for diagnosis.

In recent years, there have been many reports of the evaluation of MME by ultrasonography, and it has been found that MME on ultrasonography can show the progress of knee osteoarthritis, similar to MME evaluation by MRI [7, 10].

Though the evaluation of MME has been done previously by MRI, in the present study, MME was evaluated by ultrasonography, which provides an easy, noninvasive, and inexpensive approach. Furthermore, the evaluation with ultrasonography enables the measurement of MME during weightbearing. Other reports have shown that this method was used for medical examinations of the general population [14]. In addition, the reliability of this method has been demonstrated [11].

The first limitation of this study is that information about the knees other than MME and pain was not investigated, because the subjects were taken from the general population. In other words, knee pain due to injury or knee pain due to inflammatory diseases could not be excluded. Because the evaluation of MME was performed at a specific time point, a longitudinal study is necessary. In the future, it will be necessary to investigate the detailed causes of knee pain in the general population by adding more interview items and by evaluating the synovium with ultrasonography. In addition, by conducting such investigations, we can identify those who have knee pain or who are more likely to have knee pain at an early stage. This study demonstrated that this approach will contribute more efficiently to a longer healthy life expectancy for more persons by facilitating early intervention for their osteoarthritis.

The second limitation of this study is that the height, weight, and body mass index of the subjects were not assessed, though obesity is related to knee osteoarthritis. However, it is expected that MME increases if the subject is heavy because MME was checked during weightbearing in this study. In other words, the MME measurement may be suitable as an index of the pain of knee osteoarthritis, because the factor of weight is already included in the measured MME value.

The third limitation is that the number of subjects was relatively small. Using the results of this study, a larger-scale investigation of the relationships between MME and knee pain should be performed. By doing so, it will be possible to investigate in greater detail the relationship between past knee pain and MME, which became clear in this study. It may also help elucidate the natural relief mechanism of knee pain in osteoarthritis, and it may lead to the development of new therapies for knee osteoarthritis.

The final limitation of this study is that the reason for knee pain with large MME is not known. MME is greater with weightbearing than with nonweightbearing [10]. Therefore, during weightbearing, MME causes stretching of soft tissues around the medial joint space. This may cause the knee pain around the medial joint space during weightbearing, which is the key symptom of knee osteoarthritis. However, a tear (posterior horn in particular) of the medial meniscus, considered to be a cause of MME [15], may in itself be the cause of the knee pain.

However, from the results of the present study, it became clear that the amount of MME, which could be noninvasively investigated in several seconds with ultrasonography, was related to knee pain. Thus, it is now easy to conduct a large-scale epidemiological investigation to identify symptomatic knee osteoarthritis that should receive treatment with this method, instead of epidemiological investigations of knee osteoarthritis using only conventional imaging. In addition, the results of the current study help us better understand the mechanism of knee pain in osteoarthritis.

## 5. Conclusions

The MME cut-off value for knee pain was 4.3 mm based on ROC curve analysis, with sensitivity of 0.8451 and specificity of 0.8502.
